# Outbreak control management: Lessons from SARS‐CoV‐2 infections in 2020–2022 in Hong Kong, an international municipality with high‐frequency travelers

**DOI:** 10.1002/mco2.158

**Published:** 2022-07-25

**Authors:** Yaxiu Feng, Ching Han Young, Siu Hin Lau, Ming‐Liang He

**Affiliations:** ^1^ Department of Biomedical Sciences City University of Hong Kong Hong Kong China; ^2^ CityU Shenzhen Research Institute Nanshan, Shengzhen Guangdong China; ^3^ Cellomics International Limited Hong Kong China

**Keywords:** infection control, Omicron variant, outbreak management, SARS‐CoV‐2

## Abstract

The control management of severe acute respiratory syndrome coronavirus 2 (SARS‐CoV‐2) infections is one of the most challenges in the 21st century. By May 8th, 2022, SARS‐CoV‐2 has infected over 510 million people with 6.2 million death worldwide and over 1.2 million people with 9133 deaths in the fifth wave of infection in Hong Kong. The government responded rapidly in the early days of the 2020 outbreak, and the results were encouraging to control COVID‐19 outbreak unavailable of vaccine. The quick responses to the epidemic alerts, for example, public education and control policies, kept residents safe from infection in the city with such a high population density and large‐scale travelers. Nevertheless, the extremely high infectivity, Omicron variant infections, and the shortcomings of transmission control measures led to uncontrollable outbreak in 2022. The weak immunity groups, elderly and children, experienced a high hospitalization rate and mortality rate because of low vaccination rate. Currently, the infection is under well controlled. This study timely summarizes the challenges, policy, and lessons of SARS‐CoV‐2 outbreak control from 2020 to 2022. More importantly, the lesson and policy revealed from this study may be beneficial and applied to other cities with the outbreak of highly infectious SARS‐CoV‐2.

## Graphical abstract

It saved Hong Kong from a severe outbreak in the early stages of COVID‐19 infection by the government's rapid responses. Due to extremely high infectivity, short incubation period of Omicron variants and border policies, SARS‐CoV‐2 broke out seriously in 2022 but got quickly controlled in April. The control policies on COVID‐19 in Hong Kong are important references in other cities.

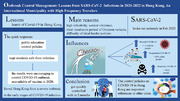

## 1 INTRODUCTION

**FIGURE 1:**
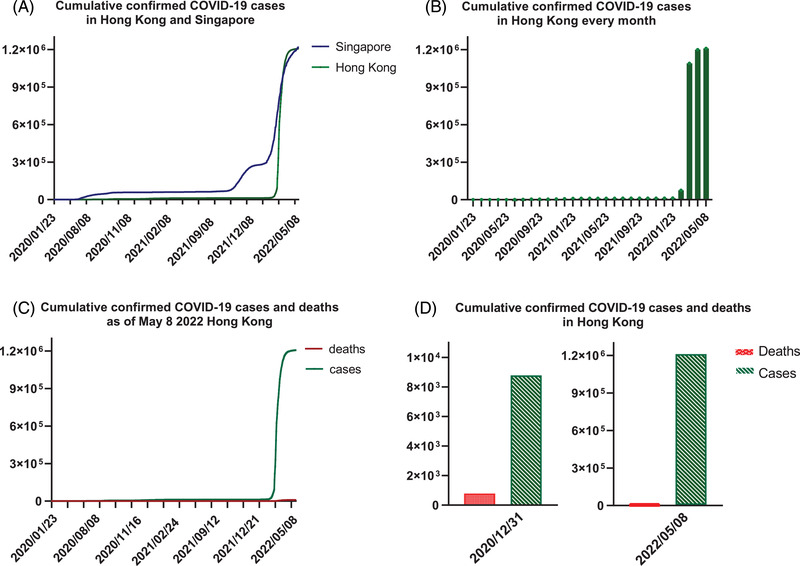
The cumulative confirmed COVID‐19 cases and deaths

In the last 2 years, Hong Kong has experienced a large‐scale outbreak of coronavirus infectious disease (COVID‐19), which was caused by a new strain of coronavirus that causes severe acute respiratory syndrome coronavirus 2 (SARS‐CoV‐2) since 2019 until now.^1^ SARS‐CoV‐2 is an enveloped virus with a 30‐kb positive‐sense (+) single‐stranded RNA genome.^2^ It is extremely infectious. The infection rate is thousand times higher than SARS‐CoV that appeared in 2003, which also had a serious impact on our society and economy.^1^, ^3^, ^4^ At the early phase of COVID‐19, the Government of the Hong Kong Special Administrative Region (HKSAR government) made a good control of the epidemic, preventive measures and transmission control policies had played important roles to manage the low infection rate in Hong Kong (https://www.chp.gov.hk/files/pdf/govt_preparedness_and_response_plan_for_novel_infectious_disease_of_public_health_significance_eng.pdf Accessed: April 30, 2022). However, the extremely high infectivity and relative low virulence of SARS‐CoV‐2 and the partial inopportune transmission controlling policies have caused a huge challenge to HKSAR government because of extensive and high frequency of traveling among Hong Kong and countries/regions worldwide.^5^ From January 2020 to present, there are more than 1.2 million cumulative confirmed SARS‐CoV‐2‐infected cases and 9133 dead cases as of May 8, 2022 (Figure 1), (https://ourworldindata.org/coronavirus Accessed: May 8, 2022), including both reverse transcription polymerase chain reaction (RT‐PCR) tests and rapid antigen test (RAT), whereas over 1.15 million are new cases amidst the fifth wave of infection from March to early May in 2022 (https://www.coronavirus.gov.hk/pdf/5th_wave_statistics/5th_wave_statistics_20220508.pdf Accessed: May 8, 2022). The severity of community outbreak has reflected the shortcomings of the infectious disease control policies.

After carefully evaluated the characteristics of Omicron variants of SARS‐CoV‐2 and many other factors, HKSAR government quickly took response to Omicron outbreak.^6^ Currently, the outbreak of SARS‐CoV‐2 is under control in Hong Kong. The positive cases were dropped from the peak over 58,000 cases/day to steadily 200–300 cases/day in May 2022 (https://ourworldindata.org/coronavirus Accessed: May 8, 2022). Because the high vaccine rate was achieved and large number of cases in the fifth wave infections, the community has established an ideal immunity barrier for SARS‐CoV‐2 infections. This study aimed to summarize the problems and improvement of the HKSAR government reactions to COVID‐19 outbreak, analyze the potential challenges that led to severe outbreak in 2022, and propose improvement directions for the next emerging infectious diseases.

## 2 THE CONTROL POLICIES AGAINST SARS‐COV‐2 PANDEMIC IN THE EARLY 2020

### 2.1 The policy and the effect after SARS‐CoV‐2 epidemic

HKSAR government made a quick response and obtained significant effects in early 2020. After receiving the novel atypical pneumonia disease outbreak alerts from China and WHO, the HKSAR government reacted quickly by issuing “serious response level” and established steering committee to manage the infectious control policies.^7^ It is to ensure proper coordination and normal operations between government departments and alert the public, and to make balance at large among health, economic needs, social behavior, and community activities. Partial boarder control was implemented instantly to screen the travelers from epidemic area by rigorous temperature measurement and fill the health declaration form to select suspected cases. The public hospitals are equipped with well‐train medical professionals and sufficient instruments to handle the suspected cases isolated from the influx of travelers.^6^, ^7^ Moreover, the supply chain of the consumables such as personal protective equipment (PPE) could be maintained for at least 3 month (https://www.legco.gov.hk/yr03‐04/english/panels/hs/papers/hs0719cb2‐3105‐4e.pdf. Accessed: May 6, 2022).

### 2.2 The reactions to the first SARS‐CoV‐2 infection case

The limitation of the partial lockdown is the breakthrough of asymptomatic patients. The first community case of SARS‐CoV‐2 infection in Hong Kong was declared in late January 2020. More aggressive border control was promptly actioned to suspend the influx of travelers from epidemic areas in China, while maintained normal supplies to Hong Kong and oversea return of Hong Kong residents.^8^ The vulnerable groups such as students were first protected by the closure of colleges and institutions through promotion of online education.^9^ Moreover, the regulations of group gathering of less than four people and the cancellation of dine‐in services at night have been enforced to eliminate the social interactions of different groups. Subsequently, working‐from‐home policies were encouraged to the enterprises to further expand the social distancing area.^8^, ^10^ In addition, the residents were encouraged to keep high standard of personal hygiene, wearing surgical masks, and frequent handwashing to prevent the spread of pathogens.

### 2.3 Follow‐up actions after community infection was discovered

Adhering to the common infectious disease management practices, the discovery of positive case in the community is followed by contact tracing, 14‐day quarantine, and clinical surveillance. In Nov 2020, the government launched a newly developed free download “LeaveHomeSafe” mobile app.^8^ It was to record the visits and leaving of the voluntary users especially in catering business and other venues in Hong Kong. The e‐system automatically can capture the close contact, send alert to them for mandatory PCR tests to screen out potential carriers and report to the authority directly. It also serves as a single‐way communication platform to update latest news of COVID‐19 by the Health Authority and Infectious Disease Control Centre in Hong Kong. The suspects with positive tests will be enforced to participate the 14‐day quarantine and clinical surveillance. To cater the large number of suspected patients, the government formed strategic teams to establish new isolation campuses and new medical laboratories.

## 3 OUTBREAK OF SARS‐COV‐2 INFECTION IN HONG KONG

Compared with other populated cities like Singapore, the number of diagnosed cases stayed relatively low in 2020–2021 (Figure 1A). In addition, the number of confirmed cases in Hong Kong had always been at a low level before the fifth wave infections (Figure 1B). Nevertheless, Hong Kong has been challenged by the largest community outbreak since Chinese New Year 2022. As of May 8, the total number of confirmed cases was more than 1.2 million and the death toll has reached 9133 (Figure 1C). Compared with the total cases up to December 31, 2020, more than 135.6 times of confirmed cases have been confirmed until May 8, 2022 (Figure 1D). The changes of extremely high infectivity and over 95% asymptomatic infections of SARS‐CoV‐2 variants (e.g., Omicron) and the not stringent enough transmission control policies might be instrumental to the uncontrollable situation.

### 3.1 High transmission efficiency of SARS‐CoV‐2

SARS‐CoV‐2 exhibits high transmissibility.^10^ It transfers from host to host by infected respiratory droplets and adheres tightly to the human upper respiratory epithelial cells that facilitate viral entry.^11^ The reproductive value (R0) of SARS‐CoV‐2 is estimated in the average of 2.5, ranging from two to eight among different mutated variants, whereas the R0 of SARS‐CoV is anticipated in the average of 2.4, ranging from 2 to 3.^12^, ^13^ The infectiousness of SARS‐CoV‐2 was peaked at averagely 5.8 days on or before the symptom's onset.^14^ Furthermore, the weak immunity groups especially elderlies are more vulnerable to SARS‐CoV‐2 infection. Although over 80% of the reported cases worldwide exhibited mild clinical presentations or even asymptomatic, the mortality rate of older adults is around 15%.^15^ The flu‐like symptoms such as fever, cough, headache, and malaise and low hospitalization rate in healthy adults had caused confusion of infection.^16^

### 3.2 The implementation of lockdown policies

Border control management is another critical strategy to prevent disease outbreak.^17^ After the announcement of pandemic of COVID‐19, many countries immediately initiated the country lockdown to limit the entry of travelers from high‐risk districts. However, Hong Kong still opens borders to accept influx from all over the world except Hubei Province. In addition, for specified categories of persons who work to maintain mandatory operations and supplies in Hong Kong, such as air crew and sea crew, and the residents return to Hong Kong from lower risk provinces of China are exempted from quarantine by special arrangement and special travelling scheme. The high connectivity of Hong Kong to other cities and the delayed response promoted the risk of importation, leading to the top three riskiest regions in China.

### 3.3 The resource coordination of medical resources between public and private sectors

Hospital resource is another key in infectious disease management. However, the shortcomings of the preparation work have been revealed by the sudden surge of confirmed cases in February 2022. It exceeded several times of the capacity to handle confirmed cases in the public hospitals (https://www.scmp.com/news/hong‐kong/health‐environment/article/3165874/coronavirus‐can‐hong‐kongs‐public‐health‐care Accessed: May 5, 2022). Patients were lining up inside and outside the emergency department as there was lack of medical resources to accept large number of patients with COVID‐19 clinical presentations.

### 3.4 Vaccination,coverage, and hesitancy

Vaccination could not only prevent infection, but also reduce the severity of the disease progression.^18^ Two types of COVID‐19 vaccines, inactivated virus vaccine (Sinovac) and the mRNA vaccine (BioNTech), were immediately launched when it was available and recommended by WHO in February 2021 (https://www.covidvaccine.gov.hk/en/. Accessed: May 8, 2022). The effective rate of the vaccines is ranged from 50% to 95%.^19^ However, it drops significantly against the Omicron variants (dominant variant in 2022) and progressively declines by time.^20^, ^21^ In addition, a Hong Kong survey reported that more than 60% of the questionnaire respondents developed vaccine hesitancy during the COVID‐19 epidemic and the hesitancy is increased following the reemergence of infection waves.^22^, ^23^ This phenomenon is highly associated with the lack of confidence on safety of the new vaccine administration, the country of vaccine production, and the vaccine efficacy.^24^, ^25^ Therefore, the vaccination rate has not been able to increase until the vaccine passport is issued. The vaccination rate of at least two doses was less than 65% in the total population in December 2021 (Table 1).

**TABLE 1:** Vaccine coverage and the COVID‐19 infected death in Hong Kong by the end of 2021

				No. of deaths with no. of vaccine doses (% of total death^a^)		
Age group	End‐2021 resident population	Received 2 doses end‐2021 (% of total^a^)	Cumulative number of deaths (% of total death)	None	1	2
<3	123,600	NA	1(0.0)	1 (100)	0	0
3–19	949,900	255,510 (26.9)	10(0.2)	6 (60.0)	4 (40.0)	0
20–29	771,900	613,586 (79.5)	10(0.2)	6 (60.0)	2 (20.0)	2 (20.0)
30–39	1,094,500	864,294 (79.0)	15(0.3)	9 (60.0)	2 (13.3)	4 (26.7)
40–49	1,160,300	963,035 (83.0)	43 (0.7)	30 (69.8)	4 (9.3)	9 (20.9)
50–59	1,191,300	924,587 (77.6)	170 (2.9)	95 (55.9)	34 (20.0)	41 (24.1)
60–69	1,122,100	679,592 (60.6)	496 (8.4)	309 (62.3)	94 (19.0)	93 (18.8)
70–79	591,300	253,378 (42.9)	977 (16.5)	625 (64.0)	201 (20.6)	151 (15.5)
≥80	398,200	71,635 (18.0)	4182 (70.8)	3036 (72.6)	728 (17.4)	418 (10.0)
**Total**	**7,403,100**	**4,625,618 (62.5)**	**5906**	**4118 (69.7)**	**1068 (18.1)**	**720 (12.2)**

^a^ Rate to the total in each age group.

(All the data are from https://www.coronavirus.gov.hk/chi/index.html#Updates_on_COVID‐19_Situation).

In the early 2022, vaccination rates increased rapidly, effectively reducing the number of deaths. The number of deaths of completed vaccination for two doses and unvaccinated group was increased from 12.2% to 69.7% (Table 1). However, the vaccination rate was declined by age. Elderly who aged 60–69, the vaccination coverage was 63%, whereas the vaccination rate was only 18% in the group aged over 80. Over 95% of the COVID‐19‐infected death cases came from the older adults aged over 60 (Table 1). Most importantly, a higher fatality rate is observed in the unvaccinated group, of which over 70% was found in the older adults group (age over 60). Yet, the death rate dropped to 11.7% for older people who received at least two doses of vaccines (Table 1). Although the wild‐type vaccines might not cover the infection of all variants of SARS‐CoV‐2, early promotion of adaptive immunity specific to COVID‐19 could significantly prevent vaccinated persons from hospitalization and increase survival rate.

### 3.5 The development of antipandemic fatigue

Apart from the regionwide border control, the HKSAR government has deployed several community social distancing strategies since the first wave of COVID‐19. For example, gathering of more than 4 people in public area is prohibited, the opening hours and seat occupancy of dine‐in catering business are limited, and compulsory lock down of entertainment premises such as gymnasium, cinemas, and bars is launched. The aggressive health‐protective measures may have sharply reduced the risk of potential transmission in the community yet developed anti‐pandemic fatigue at the same time.^26^ The long‐term restriction might have weakened the people's adherence to the government and the policies. The reemerging waves have lost the tendency of the residents to follow the infection control measures.^27^ It eventually leads to losing sight of pandemic and resumes high connectivity of social life and instrumental to the risk of community outbreak.

## 4 THE CONTROL POLICY AND ITS EFFECTS DURING THE FIFTH WAVE OUTBREAK IN HONG KONG

In the fifth wave infection, Omicron, the major SARS‐CoV‐2 variant, displays characteristics as extremely high infectivity, low virulence, and short transmission cycle (2–3 days).^28^ Therefore, the infected cases quickly increased from single digits to over 58,000 per day within 20 days that caused great challenge to medical facilities. Eventually, the total death number was greatly increased (Table 2). To raise the vaccination rate for reducing the mortality rate, the policy of vaccine pass was launched on February 24, 2022 (https://www.coronavirus.gov.hk/eng/vaccine‐pass.html. Accessed: May 8, 2022). For any person who aged 12 or above is required to be vaccinated before they enter or staying in the listed of premises, including but not limited to catering business premises, markets, shopping mall, department store, and supermarket. This policy greatly encourages the Hong Kong citizens to receive vaccination, especially the elderly (https://www.elderly.gov.hk/eindex.html Accessed: May 8, 2022). The vaccination status of age group of 60–69, 70–79, and above 80 were evolved from 63%, 45%, and 18% to 83.1%, 73.8%, and 53.3% (Table 1 and Table 2) by the early May, respectively.

**TABLE 2:** The cumulative death and associated vaccination status of different age group in Hong Kong by May 8, 2022

Age groups	End‐2021 resident population	Received two doses May 2022 (% of total^a^)	Received three doses May 2022 (% of total^a^)	Cumulative number of deaths (% of total death)	No. of deaths with no. of vaccine doses (% of total death^a^)			
					None	1	2	3
<3	123,600	NA	NA	1(0.0)	1 (100)	0	0	0
3–11	502,600	232,708 (46.3)	181 (0.0)	6 (0.1)	5 (83.3)	1 (16.7)	0	0
12–19	447,300	383,916 (85.8)	97,293 (21.8)	5 (0.1)	3 (60.0)	1 (20.0)	1 (20.0)	0
20–29	771,900	722,683 (93.6)	315,208 (40.8)	14 (0.2)	7 (50.0)	1 (7.2)	6 (42.8)	0
30–39	1,094,500	1,043,025 (95.3)	560,301 (51.2)	25 (0.3)	15 (60.0)	3 (12.0)	4 (16.0)	3 (12.0)
40–49	1,160,300	1,127,064 (97.2)	728,673 (62.8)	61 (0.6)	37 (60.6)	5 (8.2)	15 (24.6)	4 (6.6)
50–59	1,191,300	1,111,784 (93.3)	732,571 (61.5)	247 (2.7)	153 (61.9)	27 (10.9)	65 (26.3)	2 (0.8)
60–69	1,122,100	932,517 (83.1)	573,073 (51.1)	756 (8.3)	493 (65.2)	114 (15.1)	133 (17.6)	16 (2.1)
70–79	591,300	436,542 (73.8)	232,990 (39.4)	1,529 (16.7)	1037 (67.8)	258 (16.9)	213 (13.9)	21 (1.4)
≥80	398,200	212,282 (53.3)	69,364 (17.4)	6487 (71.0)	4844 (74.7)	1017 (15.7)	593 (9.1)	33 (0.5)
**Total**	**7,403,100**	**6,202521 (83.8)**	**3,309,654 (44.7)**	**9133** **^b^**	**6595 (72.2)**	**1427 (15.6)**	**1030 (11.3)**	**79 (0.9)**

^a^Rate to the total in each age group.

^b^ COVID‐19 death case is defined as a death in a person with positive SARS‐CoV‐2 result and died within 28 days of the first positive specimen collection day. The underlying cause of death may have been unrelated to COVID‐19. The total death toll of 9133 includes two pending deaths, which are available from data sources.

(All the data are from https://www.coronavirus.gov.hk/pdf/5th_wave_statistics/5th_wave_statistics_20220508.pdf).

**FIGURE 2:**
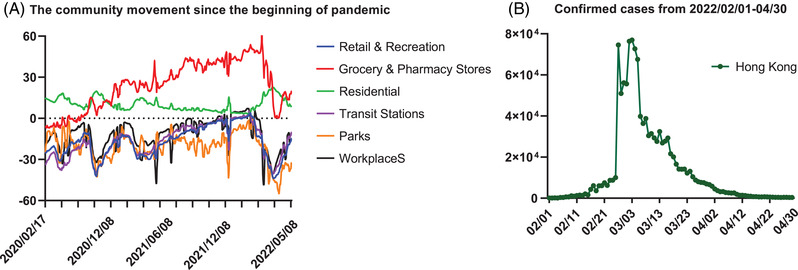
The community movement of residents and the confirmed cases during the fifth wave of COVID‐19 in Hong Kong

In addition, the government quickly responded to the fifth wave of infection by tightening the social distancing measures in January 2022 (https://hongkongfp.com/2022/01/05/breaking‐hong‐kong‐rolls‐out‐new‐covid‐social‐distancing‐rules‐dine‐in‐banned‐after‐6pm‐as‐city‐on‐verge‐of‐fifth‐wave/ Accessed: May 6, 2022). Premises including hair salons and religious premises were enforced to close to avoid group gathering. Moreover, the work‐from‐home policy was promoted again in government departments (except essential and emergency services) and local enterprises to further limit the traffic of the residents. There was a strong augment for city lockdown or more stringent gathering regulations. After carefully evaluation and balancing many factors among potential infections, social and economic cost, and health problems from the disabled health service system due to city lockdown, more stringent gathering regulations were accepted. The gathering regulation was restricted from four people to a couple of people only. Multihousehold gatherings at private premises of no more than two households were also the first time to be launched under the pandemic. The community movement in transit stations, workplaces, retail and recreation, and parks are greatly reduced since tighten the social distance measures (Figure 2A). The number of confirmed cases was quickly controlled to a low level within 2 months through the above policy measures. (Figure 2B). Currently, the outbreak is under control that is evidenced by the infection cases dropping from the peak over 58,000 per day to steadily 200–300 cases/day, and death cases dropping from 380/day to 0–1 case/day in the middle of May 2022.

## 5 BE PREPARED FOR THE NEXT EMERGING INFECTIOUS DISEASES

The exponential surge of positive COVID‐19 cases in 2022 and high vaccination rate in Hong Kong might already have led to high level of population immunity. The number of positive cases drops gradually by days. It might be hard to achieve the goal of “Zero‐COVID” (https://en.wikipedia.org/wiki/Zero‐COVID Accessed: May 7, 2022), but the reemergence of COVID‐19 or emerging of novel infectious diseases are of top concerns to prevent trauma happening again in Hong Kong.^29^

### 5.1 Transmission control management to older adults

The COVID‐19 infection and its associated mortality rates of older adults are high in Hong Kong. Vaccination coverage and the eligibility of vaccination could be a problem, but the blockage of transmission chain in their residential areas especially in the long‐term care facilities is the core factor to reduce the risk of infection among elderlies.^30^ To reduce the overcrowding issue during epidemic, the government should open a “safe campus” for less‐care intensive elderly for temporary station. The elderly who are selected to stay in the “safe campus” will be taken care by medical staffs with clinical surveillance.^31^ This action is to increase the social distancing area between individuals by eliminating the number of beds and reduce the contact risks in the nursing home. In addition, more registered nurses and medical doctors should be assigned to the nursing centers to preform necessary medical care to the elderlies to prevent travelling of the elderlies and block the transmission chain from the hospitals.

### 5.2 Novel disease and management education

During the pandemic, numerous of management issues and campaign implementation concerns have been raised by the public. The hesitancy to the government is the greatest weakness that to lose the adherence of the residents to follow the transmission control policies.^32^ Therefore, an efficient message delivery and communication channel must be built to educate the residents about novel infectious diseases, the importance of vaccines administration, and the aggressive social distancing regulations implementation. The use of digital tools can efficiently spread out the messages, avoid infodemic, and help people to understand the necessity of the policies.^33^ The community education materials should be constantly updated to the public during or after the invasion of emerging diseases.

### 5.3 Strengthen the resources allocation in public and private sectors

The outbreak in the early 2022 has revealed the deficiency of medical resources to handling large number of patients.^34^ An emergent plan under epidemic should be designed to better allocating the medical resources in public and private sectors.^8^ First of all, the government should enforce to allow more influx of the noninfectious disease's patients to the private hospitals when epidemic is incurred. It could increase flexibility of the public hospitals to manage the sudden surge of patients and group the high‐infection‐risk patients together in specified public hospitals to diminish the risk of healthcare‐associated infection in different hospitals (https://www.info.gov.hk/gia/general/202203/17/P2022031700501.htm?fontSize = 1 Accessed: May 7, 2022). Second, all medical staffs should be professionally trained and sent to support infectious diseases patients in the public hospitals during the emergent incidents. Third, a list of private clinics should be recruited to assist in triaging and treating the infected patients with mild to moderate symptoms patients to resolve the clinical burden of the public hospitals.^35^ A timely hand from different medical parties is the strongest support to the public healthcare workers to avoid the trauma happened again in Hong Kong.

## 6 FURTHER PERSPECTIVE

The challenges of infectious diseases control during COVID‐19 could be important lessons to combat the next impact of communicable diseases. The implementation of infection control policy would not only benefit Hong Kong, but also be applied to other cities in which people are suffering from highly infectious SARS‐CoV‐2 variant infections.

In the early May, the government changed the *Zero‐COVID* policy to *Dynamic Zero* policy according to the situation in Hong Kong. As an international transportation hub, Hong Kong not only needs to control the internal transmission chain, but also control external risks. Therefore, dynamic clearing is more suitable for the current environment in Hong Kong. Although it is difficult to eradicate the infection, the COVID‐19 epidemic is under control now in Hong Kong. The HKSAR government has successfully achieved the goal of “reducing deaths, reducing severe cases, and reducing infections.” At the same time, the overall protection of “key groups, key institutions, and key places” is strengthened. Such a policy has relatively little impact on Hong Kong's economy and people's lives and can be implemented for a long time. Vaccine penetration needs to continue increase. Currently, the vaccination in elders and young children are continuously promoted with enhanced government investment. Because over 90% even to 95% of vaccination rates would be achieved quickly, Hong Kong is confident to control COVID‐19 pandemic well in the future. More studies should be conducted to address the relationship between the genetic distance of SARS‐CoV‐2 mutants and infection breakthrough the vaccines. Based on updated research results, the corresponding policies should be reviewed, updated, and timely applied for more effective and cost‐effective control of SARS‐CoV‐2 infections in Hong Kong and other cities similar to Hong Kong.

## CONFLICT INTERESTS

Author M.H. is an editorial board member of MedComm. Author M.H. was not involved in the journal's review of, or decisions related to this manuscript. Author C.Y. is the employee of Cellomics International Limited, Hong Kong. The company did not provide support to the research. The other authors declared no conflict of interest.

## AUTHOR CONTRIBUTION

C.Y., Y.F., and S.L. wrote this manuscript and prepared the figures and tables. M.H. designed and supervised this project. All authors contributed to the article and approved the submitted version.

## ETHICS APPROVAL

Not applicable.

## Data Availability

The data included in this study are available upon request from the corresponding author.
